# Parallel Evolution of Tobramycin Resistance across Species and Environments

**DOI:** 10.1128/mBio.00932-20

**Published:** 2020-05-26

**Authors:** Michelle R. Scribner, Alfonso Santos-Lopez, Christopher W. Marshall, Christopher Deitrick, Vaughn S. Cooper

**Affiliations:** a Department of Microbiology and Molecular Genetics, University of Pittsburgh, Pittsburgh, Pennsylvania, USA; b Center for Evolutionary Biology and Medicine, University of Pittsburgh, Pittsburgh, Pennsylvania, USA; Geisel School of Medicine at Dartmouth

**Keywords:** *Acinetobacter*, *Pseudomonas aeruginosa*, aminoglycoside, drug resistance evolution, population genetics

## Abstract

Different species exposed to a common stress may adapt by mutations in shared pathways or in unique systems, depending on how past environments have molded their genomes. Understanding how diverse bacterial pathogens evolve in response to an antimicrobial treatment is a pressing example of this problem, where discovery of molecular parallelism could lead to clinically useful predictions. Evolution experiments with pathogens in environments containing antibiotics, combined with periodic whole-population genome sequencing, can be used to identify many contending routes to antimicrobial resistance. We separately propagated two clinically relevant Gram-negative pathogens, Pseudomonas aeruginosa and Acinetobacter baumannii, in increasing concentrations of tobramycin in two different environments each: planktonic and biofilm. Independently of the pathogen, the populations adapted to tobramycin selection by parallel evolution of mutations in *fusA1*, encoding elongation factor G, and *ptsP*, encoding phosphoenolpyruvate phosphotransferase. As neither gene is a direct target of this aminoglycoside, mutations to either are unexpected and underreported causes of resistance. Additionally, both species acquired antibiotic resistance-associated mutations that were more prevalent in the biofilm lifestyle than in the planktonic lifestyle; these mutations were in electron transport chain components in A. baumannii and lipopolysaccharide biosynthesis enzymes in P. aeruginosa populations. Using existing databases, we discovered site-specific parallelism of *fusA1* mutations that extends across bacterial phyla and clinical isolates. This study suggests that strong selective pressures, such as antibiotic treatment, may result in high levels of predictability in molecular targets of evolution, despite differences between organisms’ genetic backgrounds and environments.

## Abstract (precis)

The rise of antimicrobial resistance is a leading medical threat, motivating efforts to forecast both its evolutionary dynamics and its genetic causes. Aminoglycosides are a major class of antibiotics that disrupt translation, but resistance may occur by a number of mechanisms. Here, we show the repeated evolution of resistance to the aminoglycoside tobramycin in both P. aeruginosa and A. baumannii via mutations in *fusA1*, encoding elongation factor G, and *ptsP*, encoding the nitrogen-specific phosphotransferase system. Laboratory evolution and whole-population genome sequencing were used to identify these targets, but mutations at identical amino acid positions were also found in published genomes of diverse bacterial species and clinical isolates. We also identified other resistance mechanisms associated with growth in biofilms that likely interfere with drug binding or uptake. Characterizing the evolution of multiple species in the presence of antibiotics can identify new, repeatable causes of resistance that may be predicted and counteracted by alternative treatment.

## INTRODUCTION

The notion that evolution can be forecasted at the level of phenotype, gene, or even amino acid is no longer a fantasy in the postgenomic era (1). Most forecasting efforts rely on history to anticipate the future, and the explosive growth of whole-genome sequencing (WGS) now sets the stage to resolve evolutionary phenomena in action and determine the probabilities of the next selected path. Among the best examples, analysis by WGS of bacterial populations exposed to strong selection, like antibiotics, will likely identify genotypes that produce resistance and increased fitness (2–6). Repeated instances of selection with the same antibiotic may enrich the same types of mutations and, ultimately, enable some measure of predictability (7, 8). For instance, we can be confident that exposing different bacteria to high doses of fluoroquinolones, like ciprofloxacin, selects for substitutions in residue 83 or 87 of the drug target, DNA gyrase A (9, 10). Furthermore, drug resistance phenotypes have been predicted from genome sequence data for certain bacterial species (11, 12). These successful predictions likely result from an underlying genetic constraint, where relatively few single mutations can achieve high-level resistance, as well as population genetic power, where strong selection acts on populations with an ample mutation supply (7).

Yet predicting evolution may be hampered when these conditions are not met or when antibiotic selection produces species- or environment-specific outcomes. Evolution experiments in environments containing antibiotics have demonstrated that subjecting different bacterial strains to the same antibiotic treatment regime (13–15) or the same strains to different environments (2, 16) can select for different drug resistance levels as well as molecular targets. We can test the potential for predicting the evolved levels and genetic causes of drug resistance by studying the evolution of resistance in different environments and across different species with inherently different genetic backgrounds.

Acinetobacter baumannii and Pseudomonas aeruginosa are ESKAPE (Enterococcus faecium, Staphylococcus aureus, Klebsiella pneumoniae, Acinetobacter baumannii, Pseudomonas aeruginosa, and *Enterobacter* species) pathogens that are responsible for multidrug-resistant infections (17). These species are members of the *Moraxellaceae* and *Pseudomonadeae* families, respectively, and the genomes of the species differ in size by approximately 2.7 Mb, or more than 40%. Infections with these two opportunistic pathogens are often associated with a biofilm mode of growth (18, 19), where the bacteria grow in aggregates on surfaces and are protected from antimicrobials by a number of mechanisms. This biofilm protection may occur from secreted substances, like polysaccharides, proteins, or environmental DNA, that limit diffusion or by slowing growth and rendering the bacteria much less susceptible to an antibiotic (20, 21). Given the lifestyle differences between cells growing in a biofilm and free-living cells, we asked whether the evolution of tobramycin (TOB) resistance could proceed by different mechanisms between these two environments. TOB is an aminoglycoside antibiotic commonly used to treat infections caused by Gram-negative pathogens. Aminoglycosides are actively transported into the cell following binding to the outer membrane and can subsequently cause cell death by binding the ribosome and disrupting translation (22, 23). Resistance to aminoglycosides can occur by several mechanisms, including alteration of the translation machinery, reduced uptake, increased drug efflux, and enzymatic inactivation of the drug (24–26). However, the prevalence of these resistance strategies in different environments is incompletely understood.

Here, we experimentally propagated two bacterial species from different families, A. baumannii and P. aeruginosa, in increasing concentrations of the aminoglycoside TOB in both planktonic and biofilm environments. We performed whole-population genome sequencing at regular intervals for each lineage to identify the range of molecular mechanisms of resistance available in these species and environments. Furthermore, we aimed to examine the evolutionary dynamics of adaptation—what mutations are favored at each drug concentration in each environment—in the presence of TOB. The success of the available molecular mechanisms of resistance is determined by the order in which causative mutations occur (26), the fitness imposed by those mechanisms at a given drug concentration in the selective environment (27), and the combinations of these mutations that are selectively tolerated (28). This study depicts both marked gene- and even domain-level parallelism in the evolved genotypes as well as genetic differences between lifestyles that indicate shifts in the mode of action of TOB with potentially important clinical consequences.

## RESULTS

**FIG 1:**
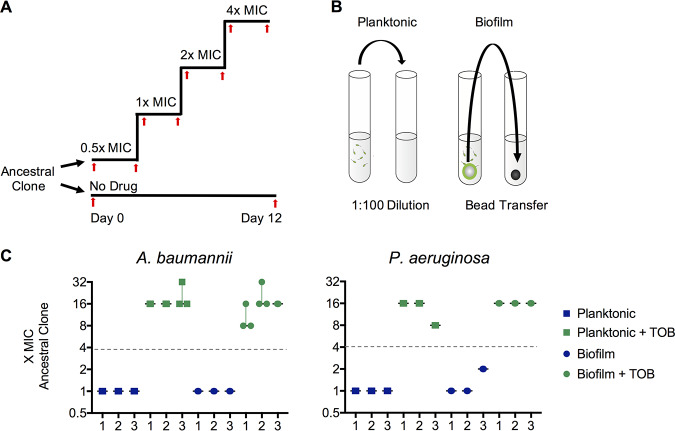
Parallel evolution of tobramycin resistance level across species and environments. Populations of A. baumannii and P. aeruginosa were propagated in minimal medium with either increasing concentrations of tobramycin or no drug and in either a planktonic or a biofilm lifestyle. Five replicate populations were propagated per treatment. (A) Populations were either propagated for 12 days in no antibiotic or inoculated into half the MIC of tobramycin, with the concentrations being doubled every 72 h. Samples of each population were archived for later phenotypic analysis and sequencing periodically throughout the experiment (red arrows). (B) Populations were propagated with selection for either planktonic growth through a daily 1:100 dilution or biofilm growth through a daily bead transfer, which forces cells to undergo the entire biofilm life cycle of attachment, growth, dispersion, and reattachment every 24 h, as described in previous work (79). (C) Tobramycin resistance level relative to that for the ancestral clone for three randomly chosen populations per treatment after 12 days of evolution. MICs were determined by microdilution in Mueller-Hinton broth according to CLSI guidelines. The fold change in the MIC for three replicates per population is shown, with the median fold change and range indicated. The A. baumannii ancestral clone MIC was 1.0 mg/liter and the P. aeruginosa ancestral clone MIC was 0.5 mg/liter in Mueller-Hinton broth. Populations had to acquire resistance to TOB at a concentration 4× the MIC for the ancestral strain in order to survive the experiment (gray dashed line).

We used TOB-sensitive ancestral clones A. baumannii ATCC 17978 and P. aeruginosa strain UCBPP-PA14 (PA14) to inoculate five replicate, single-species lineages for each of four treatments: planktonic growth without drug, planktonic growth with drug, biofilm growth without drug, and biofilm growth with drug. The evolution experiment was performed using a previously described protocol in which planktonic populations were propagated through a 1:100 dilution into fresh medium every 24 h and biofilm populations were propagated through the transfer of a colonized 7-mm polystyrene bead (16, 29, 30). This produces similar transfer sizes for both the planktonic and biofilm treatments: approximately 1 × 10^7^ CFU/transfer for A. baumannii and 2 × 10^8^ CFU/transfer for P. aeruginosa. Populations undergo approximately 6.64 generations/day, producing at least an estimated 10^6^ new mutations each day (3, 16). Populations were initially exposed to 0.5× MIC, and the antibiotic concentrations were subsequently doubled every 72 h. We propagated the populations for 12 days and periodically froze samples for later sequencing and phenotypic analysis. The experimental design is illustrated in Fig. 1A and B.

### Parallel evolution of TOB resistance phenotypes and genotypes.

For a population to survive to the end of the experiment, it must evolve resistance to at least four times the TOB concentration that would kill the ancestral clone. Previous evolution experiments in the presence of antibiotics showed that replicate populations may acquire different levels of resistance in response to the same antibiotic treatment regime when evolving in different environments (13, 16, 31). While the resistance levels did not change during the experiment for populations not exposed to antibiotics, populations propagated under TOB selection demonstrated 8× to 32× the MIC of the ancestral clone (Fig. 1C). These gains in resistance correspond to an increase from sensitive to intermediate or resistant according to Clinical and Laboratory Standards Institute (CLSI) breakpoints (32).

**FIG 2:**
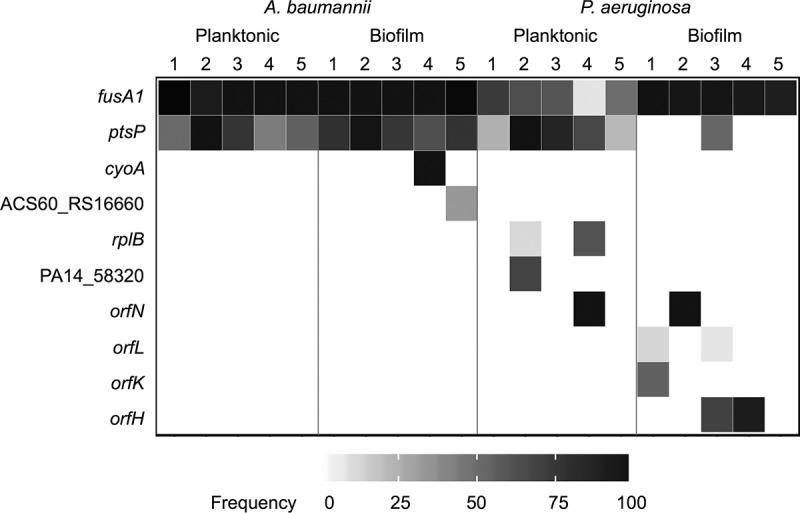
Population sequencing reveals interspecies parallelism and the lifestyle-associated molecular targets of evolution. Mutations identified by whole-population genome sequencing of tobramycin-treated populations of A. baumannii and P. aeruginosa. Five populations per treatment were sequenced after 12 days of experimental evolution. Shading indicates the total frequency of all mutations in each gene within a population at day 12.

Prior mutant screens have indicated that mutations in as many as 135 genes can produce low-level resistance to aminoglycosides, suggesting that TOB resistance may arise by mutations in diverse molecular targets (33). Instead, whole-genome sequencing of the 20 TOB-treated populations at day 12 revealed that mutations in only a few loci rose to high frequencies (Fig. 2; see also Fig. S2 in the supplemental material). The large effective population sizes (>10^7^ CFU) of these experiments ensured that mutations occurred in nearly every position across the genome and often occurred multiple times (3, 16). Further, the large sizes of these populations, despite strong drug selection, greatly empowers selection relative to the effects of drift or mutation pressure (3, 34). Therefore, mutations identified by population-wide WGS, which, in our case, reliably detects those occurring at a ≥5% frequency, represent the fittest resistant genotypes among many contenders. Mutations in the same genes selected in parallel across antibiotic-treated populations (Fig. 2) provide clear evidence of their fitness benefits in the presence of TOB, and their absence in drug-free populations indicates that they were not simply selected by other experimental conditions (Fig. S2). In the unlikely possibility that these particular loci experienced significantly higher mutation rates in the presence of TOB, only selection would have driven them to these frequencies within 3 to 12 days (3).

Despite the many differences between A. baumannii and P. aeruginosa, both species frequently acquired mutations in *fusA1* and *ptsP* (Fig. 2). The *fusA1* gene encodes elongation factor G (EF-G), an essential protein which functions in catalyzing translocation and ribosome recycling during translation (35). While A. baumannii has one copy of *fusA1*, P. aeruginosa and other *Pseudomonas* species also encode the paralogous gene *fusA2* (36). Mutations to EF-G have received little attention as a mechanism of TOB resistance in P. aeruginosa, and there are no previous reports of mutations to EF-G being a mechanism of TOB resistance in A. baumannii, to the best of our knowledge (24, 37). EF-G is also not known to be a binding target of TOB; however, this protein is the direct binding target of other antibiotics, including fusidic acid and argyrin B (38, 39). The exact mechanism by which mutations in EF-G confer aminoglycoside resistance is currently unknown, but this study demonstrates that it is an important resistance determinant. The *ptsP* gene encodes the phosphoenolpyruvate phosphotransferase protein, which is part of the nitrogen phosphotransferase system and which has been identified to be a target of TOB resistance for P. aeruginosa but not for A. baumannii, to the best of our knowledge (33, 37). The mechanism by which mutations in *ptsP* confer resistance to TOB is also unknown. However, the nitrogen phosphotransferase system has been shown to regulate the GigA and GigB pathway, which functions in coordinating the antibiotic stress response, suggesting a link between the nitrogen phosphotransferase system and coordination of the transcriptional response to antibiotic pressure (40). Therefore, even though mutations in as many as 135 genes confer reduced susceptibility, mutations in two genes, *fusA1* and *ptsP*, appear to confer the greatest fitness advantages in the presence of TOB across species and environments.

To distinguish the specific contributions of mutations in these genes to TOB resistance, we obtained isogenic mutants by isolating clones from evolved A. baumannii and P. aeruginosa populations and genotyping them by WGS. We measured by broth microdilution the TOB MICs for seven isogenic *fusA1* mutants, including A. baumannii mutants with the R59H and G460V mutations and P. aeruginosa mutants with the Q129H, N592I, F596L, Q678L, and R680C mutations (Table 1). Regardless of the species or amino acid change, *fusA1* mutations conferred an increase in TOB resistance consisting of an increase in the MIC of 2 to 4 times the MIC for the ancestor. We also measured the resistance levels of two *ptsP* mutants of P. aeruginosa (the Δ42bp mutant, in which nucleotides [nt] 1846 to 1887/2295 were deleted, and the Δ14bp mutant, in which nt 1296 to 1309/2295 were deleted) and found that these mutations produce similar increases in resistance (MICs 2 and 3 times the MIC for the ancestor, respectively). Mutants of both species with both *fusA1* and *ptsP* mutations generally exhibited higher levels of resistance to TOB than mutants with the *fusA1* or *ptsP* mutation alone (4× to 8× the MIC for the ancestral clone) at a level that is consistent with an additive effect of these two mutations on the MIC. To examine if these mutations produced increased resistance in a biofilm environment, we treated polystyrene beads colonized with *fusA1*, *ptsP*, and *fusA1 ptsP* mutants with TOB. Biofilms of the evolved mutants survived in the presence of concentrations of TOB at least 3 times higher than the concentration in the presence of which biofilms composed of the ancestral genotype survived (Table S1), suggesting that these mutations also increased TOB resistance in a biofilm environment. Mutations in *fusA1* also increased the MICs of other ribosome-targeting antibiotics, including amikacin, gentamicin, and tigecycline; however, mutations in *ptsP* did not produce cross-resistance to the other antibiotics tested (Fig. S3A), suggesting specificity to TOB resistance. While species-specific mutations did occur in several genes, as discussed below, the parallel evolution of mutations in *fusA1* and *ptsP* across A. baumannii and P. aeruginosa indicates that, regardless of genetic background, mutations to these two genes are among the few that jointly increase TOB resistance and fitness under these conditions.

**TABLE 1:** Evolved mutant clones genotyped by WGS demonstrate increased TOB resistance (MIC) relative to the ancestral clone^*a*^

Species	Genotype	MIC (mg/liter)		Fold change^*b*^
		Median	Range	
A. baumannii	Ancestor	1.0	1.0–2.0	
	*fusA1* R59H	4.0	2.0–4.0	4.0
	*fusA1* G460V	4.0	4.0–8.0	4.0
	*cyoA* V53V *ptsP* indel of nt 1742/2295 *ACX60_RS12470* A173D	4.0	2.0–4.0	4.0
	*fusA1* G460V *ptsP* indel of nt 1340–1368/2295	8.0	8.0–8.0	8.0
	*fusA1* G460V *ptsP* indel of nt 1788/2295	8.0	8.0–8.0	8.0
	*fusA1* F535L *ptsP* Δ2bp 587–588/2295 *ACX60_RS04175* R62C	8.0	8.0–16.0	8.0
P. aeruginosa	Ancestor	0.5	0.25–1.0	
	*fusA1* Q129H	1.5	1.0–2.0	3.0
	*fusA1* N592I	2.0	2.0–2.0	4.0
	*fusA1* F596L	2.0	1.0–2.0	4.0
	*fusA1* Q678L	2.0	1.0–2.0	4.0
	*fusA1* R680C	1.0	1.0–2.0	2.0
	*ptsP* Δ42bp (deletion of nt 1846–1887/2280)	1.0	1.0–2.0	2.0
	*ptsP* Δ14bp (deletion of nt 1296–1309/2280)	1.5	1.0–2.0	3.0
	*fusA1* Q678L *ptsP* R301C	4.0	1.0–4.0	8.0
	*fusA1* T456A *ptsP* V661E	2.0	1.0–4.0	4.0
	*fusA1* Q563L *ptsP* E335*	4.0	2.0–4.0	8.0
	*fusA1* R680C *ptsP* E335*	4.0	2.0–4.0	8.0
	*fusA1* T671A *ptsP* Δ1bp (deletion of nt 1122/2280)	4.0	2.0–8.0	8.0
	*fusA1* N592I *orfN* Δ1bp (deletion of nt 148/1017)	2.0	2.0–4.0	4.0

a Data are from 4 replicates.

b Fold change in the MIC from that for the ancestor.

10.1128/mBio.00932-20.6 TABLE S1 Evolved mutant clones genotyped by WGS demonstrate increased tobramycin resistance in a biofilm environment (MIC, data reflects four replicates) relative to the ancestral clone. Download Table S1, DOCX file, 0.01 MB. Copyright © 2020 Scribner et al. 2020 Scribner et al. This content is distributed under the terms of the Creative Commons Attribution 4.0 International license.

### Environment-associated adaptations to TOB selection.

Bacterial growth in different environments often involves distinct physiological processes, including nutrient uptake, metabolic pathways, growth rates, and investment in defenses (41). Consequently, the stresses produced by a given antibiotic may vary in different environments. Experimental evolution under both planktonic and biofilm conditions allows us to test if the genetic pathways of adaptation depend on the external environment. TOB selection enriched multiple mutations in *fusA1* within each population regardless of the environment, but their frequencies differed with lifestyle. For P. aeruginosa, *fusA1* mutations dominated biofilm populations in the final sample (mean ± standard deviation, 95.4% ± 3.7%), but their frequencies varied in planktonic populations (mean ± standard deviation, 50.4% ± 25.7%) (Fig. 2). Furthermore, 12 distinct mutations in *ptsP* rose to detectable frequencies in P. aeruginosa lineages, and 11 of these occurred in planktonic populations, demonstrating a strong selection for *ptsP* mutations under the planktonic growth condition that is less pronounced than that under the biofilm growth condition (Data Set S2). Meanwhile, P. aeruginosa biofilm populations frequently acquired mutations in the *orfK*, *orfH*, *orfL*, or *orfN* gene (subsequently referred to jointly as *orfKHLN*), encoding O antigen biosynthesis enzymes, and this locus was mutated in only one of the planktonic populations exposed to TOB (42, 43). A mutation in *orfK* also occurred at a low frequency in a population with no TOB selection, suggesting that these mutations may be beneficial under a variety of conditions but most beneficial in the combination of biofilm growth and TOB selection (Fig. S2). Indeed, mutants with *orfN* mutations together with *fusA1* mutations were more resistant than mutants with the *fusA1* mutation alone in the experimental medium (Fig. S3B) and increased biofilm production (Fig. S3C).

10.1128/mBio.00932-20.8 DATA SET S1 Mutations and frequencies within each population at each time point for A. baumannii. Download Data Set S1, XLSX file, 0.02 MB. Copyright © 2020 Scribner et al. 2020 Scribner et al. This content is distributed under the terms of the Creative Commons Attribution 4.0 International license.

10.1128/mBio.00932-20.9 DATA SET S2 Mutations and frequencies within each population at each time point for P. aeruginosa. Download Data Set S2, XLSX file, 0.02 MB. Copyright © 2020 Scribner et al. 2020 Scribner et al. This content is distributed under the terms of the Creative Commons Attribution 4.0 International license.

The genetic targets of resistance were more consistent in A. baumannii populations treated with TOB, with the *fusA1* and *ptsP* mutations reaching similar frequencies in both the biofilm and planktonic treatments by the end of the experiment. However, six independent mutations in *cyoA* and *cyoB* (subsequently referred to jointly as *cyoAB*), encoding components of the electron transport chain, rose to detectable frequencies prior to day 12, and all of these occurred in the biofilm lineages (Fig. 2; Fig. S4) (7). Together, the parallel evolution of mutations in *orfKHLN* and *cyoAB* in the biofilm lineages but not the planktonic lineages indicates that lifestyle may influence the strength of selection for certain TOB resistance mutations.

The parallel evolution of mutations in four genes associated with O antigen biosynthesis in P. aeruginosa suggests that selection favors alterations in the outer membrane when biofilm populations are exposed to TOB (8, 42, 44). Consistent with this observation, mutations in *orfN*, a homolog of *wbpL* in strain PAO1, have been shown to cause the loss of the O-specific antigen in the strain PA14 lipopolysaccharide (LPS), a phenotype which frequently arises during chronic P. aeruginosa infection in cystic fibrosis (CF) patients (44). This loss of O-specific antigen also increases resistance to aminoglycosides by reducing permeability and the binding affinity to the outer membrane (45, 46). Together, these reports indicate that the selected *orfKHLN* mutations may produce TOB resistance by altering the outer membrane to reduce drug binding or uptake. Similarly, populations of A. baumannii evolved with TOB and biofilm selection acquired mutations in the *cyoAB* operon. Mutations in electron transport chain components, like *cyoAB*, have previously been suggested to increase resistance to aminoglycosides by reducing membrane permeability (33, 47, 48). Therefore, although the biofilm populations of the two species propagated in this experiment evolved mutations in different loci (affecting LPS biosynthesis genes in P. aeruginosa and electron transport chain components in A. baumannii), these may represent the parallelism of a broad strategy to alter the membrane structure or permeability under combined biofilm and aminoglycoside selection.

### Population genetic dynamics of TOB resistance evolution.

**FIG 3:**
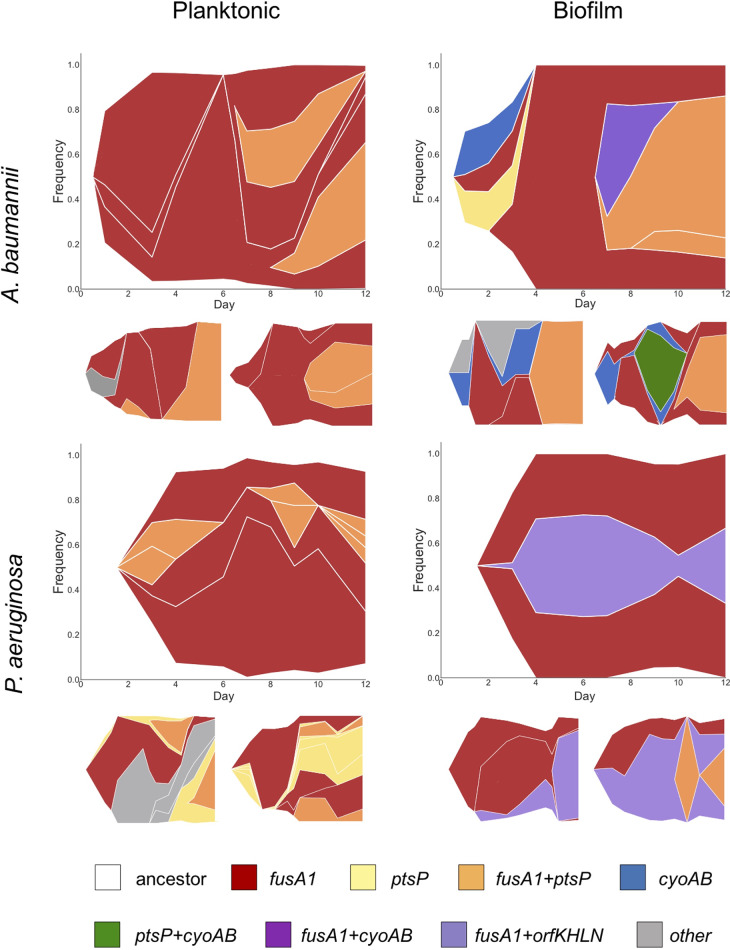
Evolutionary dynamics of bacterial populations in increasing concentrations of tobramycin. Muller plot diagrams display the genotype frequencies as a proportion of the population throughout 12 days of evolution for three populations per treatment. Genotypes are shaded by the putative driver loci that are mutated. Different lineages of the same color represent mutations at different positions within the same locus that coexist within the population. The frequency of genotypes at every time point is represented by the height of the graph that it spans at that time point. In situations in which a first mutation arises in the background of the ancestral genotype, the color representing that genotype can be seen beginning from the white background, whereas in situations in which a mutation arises in the background of another mutation, thus generating a new genotype, the new color arises in the middle of the existing genotype. Mutations occurring in the background of putative driver mutations are not shown but may be viewed in linear allele frequency plots of each population in Fig. S4 in the supplemental material.

We used longitudinal population sequencing of three lineages per treatment to determine the effects of species and the environment on the temporal dynamics of evolution in the presence of increasing concentrations of TOB. The frequencies of mutated alleles within a population were plotted over time to display mutation trajectories (Fig. S4). Next, to infer genotypes based on allele frequency data, we developed and implemented a novel set of computational tools which applies a hierarchical clustering algorithm to genetic distance metrics. The lineage of genotypes was subsequently inferred using a Bayesian approach. This application then illustrates the observed dynamics of genotypes over the course of evolution through Muller plots (Fig. 3; see Materials and Methods). Genotype frequency is represented by the breadth of shading with colors corresponding to the presence of the *fusA1*, *ptsP*, *cyoAB*, and *orfKHLN* mutations within that genotype.

In all lineages, regardless of the environment, mutations in *fusA1* were detected at either 0.5× MIC or 1.0× MIC and subsequently rose to high frequencies (Fig. 3, red). Their rapid rise in frequency in the first few days of the experiment suggests that *fusA1* mutations were the fittest contending mutations at subinhibitory concentrations of TOB. Some *fusA1* mutations, for example, *fusA1* G460V, occurred in multiple independent lineages. Given the extensive gene-level parallelism observed in these experiments, these mutations may reflect parallel evolution at the amino acid level, but we cannot exclude the possibility that these mutations were introduced at undetectable frequencies in the starting culture (Fig. S4). However, 20 distinct mutations in *fusA1* were selected in lineages treated with TOB and never detected in drug-free lineages, demonstrating that *fusA1* mutations were acquired and highly advantageous under TOB selection. Lineages with different *fusA1* single nucleotide polymorphisms (SNPs) coexisted in some populations for the duration of the experiment, and secondary mutations were frequently selected on these genotypes in the genes discussed above (*ptsP*, *orfKHLN*, and *cyoAB*; Fig. 3). A. baumannii populations tended to become dominated by *fusA1 ptsP* genotypes, but all three biofilm populations also selected *cyoAB* mutants prior to day 9 (up to 2× MIC). This *cyoAB* genotype was ultimately outcompeted by a *fusA1 ptsP* genotype at 4× MIC of TOB, indicating the superiority of the latter genotype at the higher drug concentration. This result was confirmed by comparisons of the MICs for the *cyoA ptsP* and *fusA1 ptsP* mutants, which showed that mutants with the former genotype were unable to survive in the presence of the final antibiotic concentration in the evolution experiment (Table 1). In planktonic populations of P. aeruginosa, the *fusA1*, *ptsP*, and *fusA1 ptsP* haplotypes were prevalent throughout the experiment (Fig. 3). In contrast, biofilm populations of P. aeruginosa repeatedly selected *orfKHLN* mutants on a *fusA1* background. These evolutionary dynamics demonstrate that, following the initial selection of a *fusA1* mutation, selection favored secondary mutations particular to the lifestyle and the species.

### Parallelism of aminoglycoside resistance mechanisms across species.

**FIG 4:**
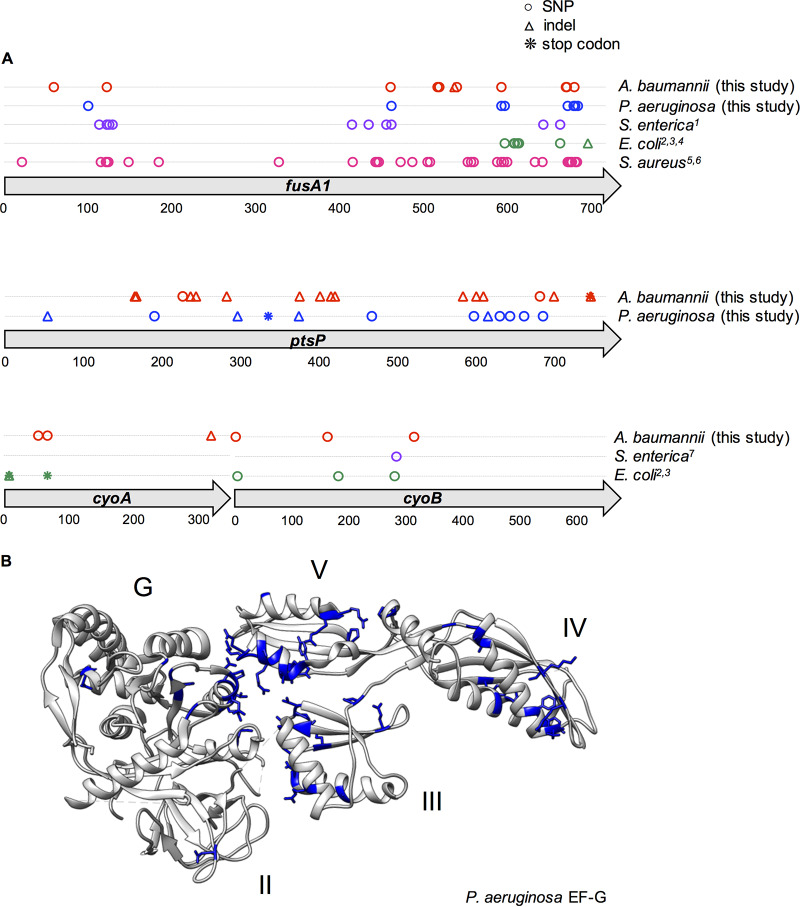
Parallelism of mutations in genetic loci associated with aminoglycoside resistance across species. (A) All mutations that occurred at any point in the experiment within the *fusA1*, *ptsP*, *cyoA*, or *cyoB* gene are indicated by a symbol at their position within the consensus amino acid sequence. Mutations reported in previous literature in other species are indicated and color coded by species; these mutations were either selected by aminoglycoside treatment *in vitro* or selected by another antibiotic and subsequently demonstrated to confer resistance to aminoglycosides. SNPs are indicated by circles, insertions or deletions (indels) are indicated by triangles, and stop codon mutations are indicated by asterisks. For each gene, the encoded amino acid sequences for all species in which mutations were identified were aligned. Mutations are shown according to their position in the resulting consensus amino acid sequence. (Top) *fusA1* gene; (middle) *ptsP* gene; (bottom) *cyoA* and *cyoB* genes. The referenced literature is identified as follows: 1, Johanson and Hughes (38); 2, Jahn et al. (49); 3, Ibacache-Quiroga et al. (7); 4, Mogre et al. (51); 5, Kim et al. (50); 6, Norström et al. (52); 7, Wistrand-Yuen et al. (26). (B) Amino acid positions that were mutated in any species are shown in blue on the protein structure of the strain PAO1 EF-G (56). Mutations are shown according to their corresponding position in the P. aeruginosa EF-G amino acid sequence. Domains G (GTPase), II, III, IV, and V are indicated.

The repeated evolution of the *fusA1* and *ptsP* mutations in both P. aeruginosa and A. baumannii suggested that these mutations may provide a general mechanism of TOB resistance across diverse species. We tested this hypothesis by searching published data sets and genomes for *fusA1*, *ptsP*, *cyoA*, and *cyoB* mutations (see Materials and Methods). Mutations in *fusA1* have been reported in several different species, including Escherichia coli, Salmonella enterica, and Staphylococcus aureus (7, 38, 49–52), and all laboratory studies reported that these mutations either arise in response to aminoglycoside selection or are a direct cause of aminoglycoside resistance (Fig. 4A). Mutations in *cyoA* and *cyoB* were also found in E. coli and S. enterica in these experiments (7, 26, 49).

We performed multiple-sequence alignments of the proteins encoded by *fusA1* (EF-G), *ptsP*, *cyoA*, and *cyoB* for all of the species that acquired mutations within each locus (Table S2). We subsequently plotted all of the identified aminoglycoside-associated mutations according to their position in the consensus sequence to examine the distribution of mutations within the proteins (Fig. 4; Fig. S5). Most mutations selected in *ptsP* were indels producing a frameshift, demonstrating that the fitness advantage conferred by these variants results from a loss of function of the product (Fig. 4). Mutations affecting cytochrome components encoded by *cyoAB* acquired frameshift, stop codon, nonsynonymous, and synonymous variants, including an unusual mutation changing the start codon from AUG to AUU (Data Set S2). In contrast, mutations in EF-G occurred at 57 distinct amino acid positions and were nearly exclusively nonsynonymous SNPs. Because *fusA1* is an essential gene (53, 54) that is highly conserved (44% amino acid identity across the five species studied and much greater similarity), these mutations must presumably both increase fitness in the presence of an aminoglycoside and maintain the essential functions of the protein.

10.1128/mBio.00932-20.7 TABLE S2 Genome name and NCBI GenBank accession numbers for genomes referenced in this study. The accession numbers and locus tags for mutations driving aminoglycoside resistance in each of the species identified in this study are indicated. The amino acid length and position within the genome are also shown. Download Table S2, DOCX file, 0.02 MB. Copyright © 2020 Scribner et al. 2020 Scribner et al. This content is distributed under the terms of the Creative Commons Attribution 4.0 International license.

The distribution of mutations within EF-G was similar across species and clustered primarily between amino acids 21 and 184 or between amino acids 327 and 695 in all species tested, with the exception of E. coli, in which mutations occurred only near the C terminus (Fig. 4; Fig. S5) (55). To test the likelihood that this clustering of mutations within EF-G could occur by chance, we divided EF-G into different bins and then used a chi-square goodness-of-fit test to determine if the observed count of mutations within bins differed from expectation based on bin size. The test was significant using three different binning strategies: binning mutations by protein domain (chi-square test value = 36.956, degrees of freedom [df] = 4, *P* = 1.839e−07), by 100-amino-acid intervals (chi-square test value = 54.175, df = 7, *P* = 2.172e−09), and by five randomly determined bins (chi-square test value = 41.957, df = 4, *P* =1.703e−08). Further, 10 amino acids of the EF-G consensus sequence repeatedly evolved mutations (at amino acids 122, 461, 472, 592, 596, 663, 671, 672, 680, and 681), revealing strong selection on particular sites of EF-G in response to aminoglycoside treatment regardless of the genetic background or growth conditions. We visualized mutated positions on the crystal structure of P. aeruginosa EF-G (Fig. 4B) (56) and identified further spatial clustering of the mutated residues. The mutations affected domains I (GTPase), III, IV, and V frequently, but occurred only once in domain II in these studies, which suggests that mutations to this region either are deleterious to the function of EF-G, do not confer an increase in resistance to aminoglycosides, or fulfill these criteria but produce variants less advantageous than those that rose to high frequencies in these experiments.

When antibiotic resistance occurs through mutations in essential gene products, one might expect that selection would favor peptide alterations that prevent drug action but that retain the core functionality of the enzyme or structure. More specifically, selection might select for mutations that alter residues that are more tolerant of variation and that vary among extant homologs rather than strictly conserved residues. We tested whether mutations in EF-G occurred less frequently in amino acid positions that were identical across species. On the contrary, SNPs occurred at positions with identical amino acids across species more frequently than expected by chance for EF-G (Fig. S5) (*P* = 8.002e−05, Fisher’s exact test). The mechanism by which these mutations produce aminoglycoside resistance without disrupting the essential functions of EF-G merits further study. Regardless, the parallel evolution of *fusA1* mutations across species and environments demonstrates the potential utility of this gene as a predictive marker of aminoglycoside resistance.

To examine if the precise mutations found in our *in vitro* study also arise in clinical isolates, we searched published genomes of P. aeruginosa clinical isolates from patients who had likely been treated with aminoglycosides, like TOB (24, 57–60). Mutations within *fusA1* and *ptsP* were reported in these genomes, suggesting that these mutations evolve during infections (Table 2). Although it was not possible to distinguish aminoglycoside selection as the driver of these mutations in a clinical setting, the selection of mutations at identical residues in our evolution experiment suggests that these mutations have the potential to produce the same increase in aminoglycoside resistance *in vivo*. Taken together, the parallel evolution of mutations in these genes in clinical isolates suggests that they may contribute to aminoglycoside resistance in clinically relevant genetic backgrounds and environments, including infections of the cystic fibrosis respiratory tract.

**TABLE 2:** Data sets of P. aeruginosa clinical isolates were analyzed to reveal gene and residue-level parallelism within the aminoglycoside resistance loci observed in this study

Study authors (reference)	Variants	
	*fusA1*	*ptsP*
López-Causapé et al. (58)	V93A, K430E, N482S, K504E, Y552C, P554L, D588G, P618L, T671I^*a*^	
Markussen et al. (60)	G118S, D467G	L42L, R61H
Chung et al. (57)	A418T, V538A, G610V, Q678R^*a*^	
Bolard et al. (24)	V93A, A555E, T671A^*a*^	

a Mutations at this amino acid position occurred in populations treated with TOB in this study.

## DISCUSSION

The rapidly intensifying problem of antimicrobial resistance demands an understanding of how antibiotic resistance evolves and which types of mutations or mobile elements are common causes (61, 62). Genetic screens of mutant collections have revealed potential resistance mechanisms (33), and more recently, evolve-and-resequence experiments have been used to identify the resistance mutations that produce the greatest fitness advantages under a given condition (8, 16, 37). However, the broader clinical utility of these screens for predicting the evolution of antibiotic resistance depends upon the relevance of the findings in other strains, species, or environments. This study served the dual purpose of identifying mutations that contribute to TOB resistance in A. baumannii and P. aeruginosa and demonstrating the effects of different environments and species histories on the evolutionary dynamics and causes of resistance.

In spite of the many genetic differences between A. baumannii and P. aeruginosa (the genome of the latter is much larger and contains dozens of additional putative resistance loci), we identified parallel mutations in *fusA1* and *ptsP* following tobramycin selection, a largely unknown combination of mutations conferring high levels of fitness and resistance. Furthermore, we found amino acid-level parallelism of *fusA1* mutations associated with aminoglycoside resistance, including kanamycin, gentamicin, and amikacin resistance, across diverse species, including E. coli, S. enterica, and S. aureus (Fig. 4; see also Fig. S5 in the supplemental material). While specific molecular targets of resistance shared by multiple species, for example, DNA gyrase mutations in response to fluoroquinolones, are known (8), the level of predictability of *fusA1* mutations has yet to be appreciated as an example of this phenomenon. We report that *fusA1* mutations not only arise during aminoglycoside treatment in at least five diverse species but also produce resistance to nearly every antibiotic within this clinically useful family of drugs (25). Furthermore, the level of parallelism detected distinguishes this study from merely episodic discoveries of *fusA1* mutations in multiple species. On the contrary, we found at least one *fusA1* mutation in every TOB-treated lineage in our study, making these mutations both widely available and highly predictable. The locations of mutations within EF-G revealed that the distribution of mutations was also similar across species (Fig. 4), but unlike other resistance targets that frequently acquire mutations in specific sites or narrow regions (10, 63, 64), the EF-G mutations were distributed across the length of the protein (Fig. 4). Alterations in EF-G have been suggested to produce structural changes that could interfere with aminoglycoside binding to the ribosome (24), but the mechanism(s) by which mutations across much of the protein all produce aminoglycoside resistance and how they alter protein function merit further study. The finding that *fusA1* mutations repeatedly evolve in clinical P. aeruginosa isolates from different strains and under different host conditions further supports the notion that these mutations are selected in diverse genetic backgrounds and environments, and we predict that, following antibiotic therapy, *fusA1* mutations may be considerably more prevalent than was previously appreciated. Mutations in *fusA1* also produced cross-resistance to other ribosome-targeting antibiotics in P. aeruginosa, a concerning finding, given the frequent use of tobramycin in treating infections of the CF airway (Fig. S3) (65).

Bacterial growth in biofilms also demonstrably altered the targets of TOB selection from those in well-mixed cultures in ways that motivate studies of the predominant mechanism of aminoglycoside killing and resistance when bacteria grow on surfaces or in aggregates. The selection of mutations in LPS biosynthesis genes (*orfKHLN*) and electron transport chain components (*cyoAB*) primarily in biofilm populations indicates that resistance may have resulted from altered TOB binding or uptake in this lifestyle. In addition to tobramycin’s mode of killing by disrupting translation, it has also been shown to exhibit a second, translation-independent killing mechanism by binding to the membrane (22, 46). Furthermore, aminoglycosides have been shown to induce cell killing in various types of nonreplicating bacteria (66). The enrichment of resistance mechanisms that alter drug binding or uptake in biofilm lineages may imply that the slow-growing, sessile cells in a biofilm experience weaker selection for mutations preserving translation but an increased demand to prevent tobramycin binding and uptake. More generally, these lifestyle distinctions in resistance traits suggest that the environment may influence the evolutionary dynamics of antimicrobial resistance, in concordance with the findings of previous studies (2, 16, 31). Therefore, while mutations in genes like *fusA1* represent mechanisms of resistance that are robust across a wide range of species, environments, and host conditions, considering the prevailing mode of bacterial growth may also improve predictions of other genetic causes of antimicrobial resistance.

The extent of parallelism in molecular evolution in these experiments is surprising, and its causes warrant consideration. Why would widely different species evolve to resist tobramycin by *fusA1* mutations and, to a lesser extent, *ptsP* mutations when many causes of aminoglycoside resistance are likely available (33)? Several possible explanations that are not mutually exclusive exist. One possibility is that these genes possess a high local mutation rate and thus acquire mutations more rapidly than other available molecular targets of resistance. However, it is doubtful that these mutations are more available than others, since neither was enriched in studies of mutations accumulated in the near absence of selection, nor are these mutations in loci in genome regions shown to have higher mutation rates (67, 68). Another possibility is that the target sizes of *fusA1* and *ptsP* (and, to a lesser extent, *orfKHLN* and *cyoAB*), in which multiple nonsynonymous mutations produce resistance, increase the likelihood of gene-level parallelism. More likely still is the possibility that mutations in *fusA1* and *ptsP* produce the greatest fitness benefit under these conditions and that these fitness benefits are robust to different species and environments. Other drugs may have a wider range of targets that produce the same level of fitness benefit, resulting in less parallelism.

It is notable that while *fusA1* and *ptsP* are not direct targets of TOB or other aminoglycosides, the effect of mutations in these enzymes may be conserved across species and is relatively insensitive to the genetic background. Many have appreciated that epistatic interactions can limit the parallel evolution of resistance mechanisms (69–72) and, hence, predictability (73, 74). The gene- and nucleotide-level parallelism of molecular targets of aminoglycoside resistance across species, environments, and clinical isolates holds promise for predicting molecular evolution, improving diagnostics for resistance, and informing more rational treatment design.

## MATERIALS AND METHODS

### Strains and media.

Pseudomonas aeruginosa strain UCBPP-PA14 and Acinetobacter baumannii strain ATCC 17978 were the ancestral strains used in the evolution experiments (75–77). A. baumannii ATCC 17978 was propagated for 10 days in minimal medium to preadapt it to the medium conditions prior to the evolution experiment. The minimal medium used in the evolution experiments consisted of an M9 salt base (0.1 mM CaCl_2_, 1.0 mM MgSO_4_, 42.2 mM Na_2_HPO_4_, 22 mM KH_2_PO_4_, 21.7 mM NaCl, 18.7 mM NH_4_Cl), 11.1 mM glucose, 20-ml/liter minimal essential medium (MEM) essential amino acid solution and 10-ml/liter MEM nonessential amino acid solution (catalog numbers 11130051 and 11140050; Thermo Fisher), and 1 ml/liter each of trace elements A, B, and C (catalog numbers 99182CL, 99175CL, and 99176CL; Corning). In addition, dl-lactate (catalog number 72-17-3; Sigma-Aldrich) was added to the P. aeruginosa medium to a final concentration of 10 mM in order to generate the approximate nutrient concentrations present in the cystic fibrosis lung environment (78). All cultures were grown in 18- by 150-mm glass tubes containing 5 ml of minimal medium and incubated at 37°C in a roller drum (30 rpm).

### Evolution experiment.

Evolution experiments in both P. aeruginosa and A. baumannii were initiated using a single ancestral clone. For P. aeruginosa, a single colony was selected, resuspended in phosphate-buffered saline (PBS), and then used to inoculate 20 replicate lineages. For A. baumannii, a single colony was selected, grown in minimal medium with no antibiotic for 24 h, and then used to inoculate 20 replicate lineages. The lineages were propagated with either increasing concentrations of TOB or no TOB and either planktonic or biofilm selection, such that five replicate lineages each were propagated for four experimental conditions (planktonic growth without TOB, planktonic growth with TOB, biofilm growth without TOB, and biofilm growth with TOB) for each organism. Lineages with planktonic selection were propagated through a 1:100 dilution every 24 h (50 μl in 5 ml of fresh minimal medium), and lineages with biofilm selection were propagated by transferring a colonized polystyrene bead (Cospheric, Santa Barbara, CA) to a tube of fresh medium and three fresh beads every 24 h, as described previously (78, 79). P. aeruginosa biofilm transfers were performed by transferring a bead directly to the next day’s tube, whereas A. baumannii biofilm transfers were performed by first rinsing the bead by transferring it to a tube of PBS and then transferring the bead to the next day’s tube. Lineages propagated with antibiotic selection were treated with tobramycin sulfate (Alfa Aesar, Wardhill, MA) starting at 0.5× MIC for the ancestral strain in the experimental minimal medium (0.5 mg/liter for A. baumannii and 2.0 mg/liter for P. aeruginosa), with doubling of the concentration every 72 h. The experiment was performed for 12 days, with samples collected on days 3, 4, 6, 7, 9, 10, and 12 and frozen at −80°C in either 25% glycerol for P. aeruginosa or 9% dimethyl sulfoxide for A. baumannii. Planktonic lineages were sampled by freezing an aliquot of the liquid culture, and biofilm lineages were sampled by sonicating a bead in PBS and freezing an aliquot of the resuspended cells.

### MIC assays.

We determined MICs by broth microdilution in Mueller-Hinton broth according to Clinical and Laboratory Standards Institute guidelines (32). To measure the MICs for evolved populations, we revived frozen populations by streaking of the populations onto a tryptic soy agar plate, resuspended a portion of the resulting bacterial lawn in PBS, and diluted the suspension to a 0.5 McFarland standard. We inoculated the suspension into round-bottom 96-well plates containing 2-fold dilutions of TOB at a final concentration of 5 × 10^5^ CFU/ml. The P. aeruginosa and A. baumannii MIC assay mixtures were then incubated at 37°C for 16 to 20 h or 18 to 22 h, respectively, and then the MIC was determined as the concentration in the first well that showed no growth. At least three assays were performed for each population. In P. aeruginosa, the MICs of TOB differed between when they were measured in Mueller-Hinton broth and when they were measured in the experimental minimal medium, but they reflected similar fold changes in the MIC relative to the MIC for the ancestral clone (see Fig. S1 in the supplemental material). The MICs for the clones were measured by the same procedure, with the exception that freezer stocks were streaked for isolation and MIC assays were performed using an isolated colony. The MICs of other ribosome-targeting antibiotics for the *fusA1* and *ptsP* isogenic mutants were determined using Sensititre plates according to the manufacturer’s specifications (Sensititre GN3F; Trek Diagnostics Inc., Westlake, OH).

10.1128/mBio.00932-20.1 FIG S1 MICs for P. aeruginosa evolved populations on day 12 in the minimal medium used in the evolution experiment (left) and Mueller-Hinton broth (right). MICs were determined according to CLSI guidelines, with the exception of the media used. The medians from at least three replicate MIC assays per population are shown. Download FIG S1, PDF file, 0.04 MB. Copyright © 2020 Scribner et al. 2020 Scribner et al. This content is distributed under the terms of the Creative Commons Attribution 4.0 International license.

10.1128/mBio.00932-20.2 FIG S2 Population sequencing reveals molecular adaptations to tobramycin and biofilm selection. Three populations per treatment for lineages not treated with antibiotic and five populations per treatment for lineages treated with antibiotic were sequenced at the day 12 time point for each species. Shading indicates the total frequency of the mutations in each locus at day 12. SNPs and stop codon mutations are denoted by the gene name or locus tag, amino acid change, and then the nucleotide change in parentheses. Indels are indicated by the gene name or locus tag, followed by “indel” and the nucleotide position of the indel within the affected gene. Download FIG S2, PDF file, 0.1 MB. Copyright © 2020 Scribner et al. 2020 Scribner et al. This content is distributed under the terms of the Creative Commons Attribution 4.0 International license.

10.1128/mBio.00932-20.3 FIG S3 MICs and biofilm production of P. aeruginosa *fusA1*, *fusA1 orfN*, and *ptsP* mutants compared to those of the clone with the ancestral genotype. (A) The change in the MIC of ribosome-targeting antibiotics for mutants isolated from evolved populations was determined in Mueller-Hinton broth using Sensititre plates. Shading indicates the fold increase in the MIC relative to that for the ancestral clone based on the median from three replicates. The MICs for the ancestral clone were 8 mg/liter for amikacin (AMI), 2 mg/liter for gentamicin (GEN), and 1 mg/liter for tigecycline (TGC). (B) The MICs of TOB for mutants isolated from evolved populations were determined by macrodilution using the experimental minimal medium to mimic the conditions of the evolution experiment. The medians from four replicate assays are shown. (C) Biofilm production was measured by a crystal violet biofilm assay. The means from at least four replicates are shown, and error bars represent 95% confidence intervals. *fusA1* N592I *orfN* Δ1bp corresponds to a genotype with a *fusA1* N592I mutation and a deletion at nucleotide 148/1017 of *orfN*. *ptsP* Δ14bp corresponds to a genotype with a deletion from nucleotides 1296 to 1309/2280 of the *ptsP* gene, and *ptsP* Δ42bp corresponds to a genotype with a deletion from nucleotides 1846 to 1887/2280 of the *ptsP* gene. Download FIG S3, PDF file, 0.1 MB. Copyright © 2020 Scribner et al. 2020 Scribner et al. This content is distributed under the terms of the Creative Commons Attribution 4.0 International license.

10.1128/mBio.00932-20.4 FIG S4 Allele frequency plots of three populations from each treatment. A. baumannii (top) and P. aeruginosa (bottom) mutations are plotted by their frequency within each population on each day of the experiment in which population sequencing was performed. SNPs are indicated by the gene name or locus tag, followed by the amino acid change. Insertions or deletions are indicated by the gene name or locus tag, followed by “indel” and the nucleotide position of the indel within the gene in parentheses. Download FIG S4, PDF file, 0.1 MB. Copyright © 2020 Scribner et al. 2020 Scribner et al. This content is distributed under the terms of the Creative Commons Attribution 4.0 International license.

10.1128/mBio.00932-20.5 FIG S5 Multiple-sequence alignment of the proteins encoded by *fusA1*, *ptsP*, *cyoA*, and *cyoB* for the species in which mutations have been demonstrated to confer aminoglycoside resistance. Positions with identical amino acid identities across all species are highlighted in gray. SNPs identified in this experiment and previous *in vitro* experiments are shown at their relative positions within the consensus amino acid sequence. SNPs in conserved positions are highlighted in green, and SNPs in nonconserved positions are highlighted in blue. SNPs occur in conserved positions more frequently than expected, based on the frequency of conserved positions in *fusA1* (*P* = 0.00196, Fisher’s exact test [FET]). Download FIG S5, PDF file, 0.2 MB. Copyright © 2020 Scribner et al. 2020 Scribner et al. This content is distributed under the terms of the Creative Commons Attribution 4.0 International license.

Although mutations to *orfKHLN* were frequent in the tobramycin-treated populations, only a small increase in the MIC was observed by the microdilution method in Mueller-Hinton broth. We therefore measured the MICs of isogenic *fusA1* and *fusA1 orfN* mutants by macrodilution in minimal medium to better represent the conditions of the evolution experiment. Twofold dilutions of TOB in 5 ml of minimal medium were inoculated with 5 × 10^5^ CFU/ml for each genotype, the cultures were incubated for 18 h, and the MIC was determined from the well with the lowest concentration with no visible growth.

The TOB MIC for the evolved clones in a biofilm environment was measured by inoculating tubes containing 5 ml of Mueller-Hinton broth and 5 polystyrene beads with each isogenic mutant and incubating for 24 h. Each colonized bead was then transferred to a fresh tube of Mueller-Hinton broth at 2-fold dilutions of TOB and treated for 18 h. The beads were transferred to 1 ml PBS and sonicated, and then 10 μl was transferred to 100 μl of fresh medium and incubated for 24 h. The MIC was reported as the lowest concentration of TOB at which no growth occurred (optical density at 600 nm [OD_600_] < 0.05) after this 24-h incubation. This MIC reflects the lowest TOB concentration that produced effective killing of the biofilm. MIC is reported as the median value from at least three replicates in all MIC experiments.

### Biofilm assays.

The biofilm production of mutants isolated from evolved populations was determined by a crystal violet assay using a previously described protocol (80). Overnight cultures of the mutants in the experimental minimal medium were diluted 1:100 in fresh minimal medium to a volume of 200 μl in a 96-well dish. The plates were incubated for 24 h at 37°C and then rinsed twice with water. The wells were stained with 250 μl of 0.1% crystal violet, and the plates incubated for 15 min, rinsed three times with water, and then allowed to dry overnight. Crystal violet was solubilized by adding 250 μl 95% ethanol (EtOH) solution (95% EtOH, 4.95% distilled H_2_O, 0.05% Triton X-100) to each well for 15 min. Biofilm formation was then visualized by measuring the OD_600_. The results are the averages from four replicates.

### Genome sequencing and analysis.

Whole populations were sequenced periodically throughout the experiment. For TOB-treated lineages, all populations were sequenced on day 12, and 3 lineages from each of the planktonic and biofilm conditions were also sequenced on days 3, 4, 6, 7, 8, 9, and 10 for P. aeruginosa and days 1, 3, 4, 6, 7, 9, and 10 for A. baumannii. Three no-TOB lineages from each of the biofilm and planktonic conditions were also sequenced on days 6 and 12 for P. aeruginosa and days 1, 4, 9, and 12 for A. baumannii.

Populations were prepared for sequencing by inoculating freezer stocks of the bacterial populations into the same medium and the same antibiotic concentration in which the population was growing at the time of freezing. Growth conditions identical to the population’s growth conditions at the time of freezing were maintained in order to minimize bias in the population structure during the outgrowth process. After 24 h of growth, populations were sampled by either removing an aliquot of the culture for planktonic populations or transferring beads to PBS, sonicating, and removing an aliquot of the resuspended cells for biofilm populations. DNA was extracted using a DNeasy blood and tissue kit (Qiagen, Hilden, Germany). The sequencing library was prepared as described by Turner and colleagues (30), according to a previously described protocol (81) using an Illumina Nextera kit (Illumina Inc., San Diego, CA), and sequenced using an Illumina NextSeq500 sequencer. Samples were sequenced to 160× coverage, on average, for P. aeruginosa populations and 309× coverage, on average, for A. baumannii populations.

Sequences were trimmed using Trimmomatic (v0.36) with the following criteria: leading, 20; trailing, 20; slidingwindow, 4:20; minlen,70 (82). Breseq (v0.31.0) was used to call variants using the default parameters and the -p flag when analyzing population sequences (83). These parameters call mutations only if they are present within the population at a frequency of at least 5% and are in at least 2 reads from each strand. The A. baumannii ATCC 17978-mff genome (GenBank accession number NZ_CP012004) and plasmid (GenBank accession number NZ_CP012005) sequences were downloaded from the RefSeq database. Two additional plasmids (GenBank accession numbers CP000522 and CP000523) were found to exist in our working strain and were added to this reference genome. The P. aeruginosa UCBPP-PA14 genome was downloaded from the RefSeq database (GenBank accession number NC_008463). Mutations were removed if they were also found in the ancestor’s sequence when mapped to the reference genome. Mutations that did not reach a cumulative frequency of at least 25% across all populations at all time points were removed, and mutations were also manually curated to remove biologically implausible mutations. A mutation was determined to be biologically implausible if it occurred either (i) at trajectories that were not possible given the trajectories of the putative driver mutations or (ii) only at the ends of reads, only in reads with many other mutations, or only at a low coverage (<10 reads), indicating poor read mapping at that region. When high-quality mutations in loci related to the putative driver loci or ribosome machinery were reported in New Junction Evidence by breseq, these mutations were also included in the analysis. Mutations fitting these criteria included mutations to 23S rRNA in P. aeruginosa and mutations to *ptsP*, phosphocarrier protein HPr, and NADH quinone oxidoreductase in A. baumannii. Filtering, allele frequencies, and plotting were done in R software (v3.5.3; www.r-project.org) with the packages ggplot2 (v2.2.1; https://CRAN.R-project.org/package=ggplot2) and tidyr (https://CRAN.R-project.org/package=tidyr). Muller plots were generated by C.D. using the lolipop package (v0.5.2; https://github.com/cdeitrick/lolipop) and the default parameters. These scripts predict genotypes and lineages based on the trajectories of mutations over time using a hierarchical clustering method and implement filtering criteria to eliminate singletons that do not comprise prevalent genotypes. Muller plots were manually color coded by the presence of putative driver mutations within each genotype. Additional mutations that occurred on the background of putative driver mutations can be viewed in the allele frequency plots but are not shown in Muller plots (Fig. 3; Fig. S4).

### Resistance locus alignment and mutation mapping.

Mutations in putative resistance loci (*fusA1*, *ptsP*, *cyoA*, and *cyoB*) were identified in previous literature reporting that these mutations arose in response to aminoglycoside selection or directly conferred an increase in aminoglycoside resistance by an MIC assay. The amino acid sequences of the encoded proteins for these species were obtained by searching the Features section of PATRIC for these genes in the genomes specified in these experiments (55). The amino acid sequences were aligned in PATRIC, and the mutations reported in each study were visualized according to the corresponding position in the sequence alignment (Fig. 4). We tested whether the SNPs identified in each of these genes occur in conserved positions more frequently than expected, considering the frequency of conserved positions within the genes, using a Fisher exact test (Fig. S5). The protein structure of EF-G of P. aeruginosa strain PAO1 (PDB accession number 4FN5) was obtained from the Protein Data Bank (PDB) (56, 84). All mutations associated with aminoglycoside resistance were colored blue, according to the relative position of the mutation in the P. aeruginosa EF-G protein sequence, using UCSF Chimera software (Fig. 4B) (85). Mutations in these genes in clinical isolates were found by searching for whole-genome sequencing studies of P. aeruginosa isolates from cystic fibrosis patients that reported these mutations. Studies that identified *fusA1* mutations in clinical isolates and reported increased aminoglycoside resistance *in vitro* for mutants with identical SNP changes were also included (24).

### Data availability.

All sequencing reads were deposited in NCBI under BioProject accession numbers PRJNA595915 and PRJNA485123. Detailed methods regarding data processing can be found at https://github.com/michellescribner/tobramycin_analysis_code.
