# Supplementary material for: Parallel Evolution of Tobramycin Resistance across Species and Environments
Source: mBio. 2020 May 26;11(3):e00932-20. doi: 10.1128/mBio.00932-20 (PMC7251211; doi:10.1128/mBio.00932-20)
Supplement: TABLE S1 [file mBio.00932-20-st001.docx]

| **Species** | **Genotype** | **Median MIC (mg/L)** | **Range** | **Fold Change** |
| --- | --- | --- | --- | --- |
| *A. baumannii* | Ancestor | ≤16 | 16-16 | - |
|  | *fusA1* G460V | 96 | 32-128 | ≥6 |
|  | *fusA1* G460V + *ptsP* indel 1788/2295nt | 48 | 32-64 | ≥3 |
| *P.aeruginosa* | Ancestor | ≤16 | 16-16 | - |
|  | *fusA1* N592I | 96 | 32-128 | ≥6 |
|  | *ptsP* Δ42bp 1846-1887/2280nt | 64 | 64-64 | ≥4 |
|  | *fusA1* R680C + ptsP E335* | 64 | 32-64 | ≥4 |
|  | *fusA1* N592I *+ orfN* Δ1bp 148/1017nt | 64 | 32-128 | ≥4 |

Table S1. Evolved mutant clones genotyped by WGS demonstrate increased tobramycin resistance in a biofilm environment (MIC, n=4) relative to the ancestral clone.
