# Supplementary material for: Parallel Evolution of Tobramycin Resistance across Species and Environments
Source: mBio. 2020 May 26;11(3):e00932-20. doi: 10.1128/mBio.00932-20 (PMC7251211; doi:10.1128/mBio.00932-20)
Supplement: TABLE S2 [file mBio.00932-20-st002.docx]

**Table S2.** Genome name and NCBI ID’s for genomes referenced in this study. Accession numbers and locus tags for driver mutations of aminoglycoside resistance identified in this study for each of these species are indicated. Amino acid length and position within the genome are also shown.

| Genome Name | NCBI Reference Genome ID | Gene | Product | RefSeq  Locus Tag | Position  Start | Position End | AA Length | Non-redundant protein accession number |
| --- | --- | --- | --- | --- | --- | --- | --- | --- |
| *Pseudomonas aeruginosa* UCBPP-PA14 | NC_008463 | *fusA1* | Translation elongation factor G | PA14_08820 | 755665 | 757785 | 706 | WP_003093741 |
|  |  | *ptsP* | Phosphocarrier protein kinase/phosphorylase, nitrogen regulation associated | PA14_04410 | 392770 | 395049 | 759 | WP_003084404 |
|  |  | *cyoB* | Cytochrome O ubiquinol oxidase subunit I | PA14_47190 | 4203575 | 4205551 | 658 | WP_003082705 |
|  |  | *cyoA* | Cytochrome O ubiquinol oxidase subunit II | PA14_47210 | 4205558 | 4206553 | 331 | WP_003086856 |
| *Acinetobacter baumannii* strain ATCC 17978-mff | CP012004 | *fusA* | Translation elongation factor G | ACX60_14045 | 2962952 | 2965090 | 712 | WP_005229047 |
|  |  | *ptsP* | Phosphocarrier protein kinase/phosphorylase, nitrogen regulation associated | ACX60_16055 | 3393072 | 3395366 | 764 | WP_005133238 |
|  |  | *cyoB* | Cytochrome O ubiquinol oxidase subunit I | ACX60_06730 | 1439111 | 1441102 | 663 | WP_002119744 |
|  |  | *cyoA* | Cytochrome O ubiquinol oxidase subunit II | ACX60_06735 | 1441106 | 1442158 | 350 | WP_005215377 |
| *Escherichia coli* str. K-12 substr. MG1655 | NC_000913 | *fusA* | Translation elongation factor G | b3340 | 3469422 | 3471536 | 704 | WP_000124700 |
|  |  | *cyoB* | Cytochrome O ubiquinol oxidase subunit I | b0431 | 447874 | 449865 | 663 | WP_000467180 |
|  |  | *cyoA* | Cytochrome O ubiquinol oxidase subunit II | b0432 | 449887 | 450774 | 295 | WP_001239436 |
| *Salmonella enterica* serovar Typhimurium str. LT2 | NC_003197 | *fusA* | Translation elongation factor G | STM3446 | 3599558 | 3601672 | 704 | WP_000124693 |
|  |  | *cyoB* | Cytochrome O ubiquinol oxidase subunit I | STM0442 | 495189 | 497180 | 663 | WP_000467158 |
|  |  | *cyoA* | Cytochrome O ubiquinol oxidase subunit II | STM0443 | 497191 | 498120 | 309 | WP_001239449 |
| *Staphylococcus aureus* NCTC 8325 strain | LS483365 | *fusA* | Translation elongation factor G | NCTC8325_00491 | 540862 | 542943 | 693 | WP_001788222 |
