# Supplementary figures and images for: Parallel Evolution of Tobramycin Resistance across Species and Environments
Source: mBio. 2020 May 26;11(3):e00932-20. doi: 10.1128/mBio.00932-20 (PMC7251211; doi:10.1128/mBio.00932-20)

## Minimal Media

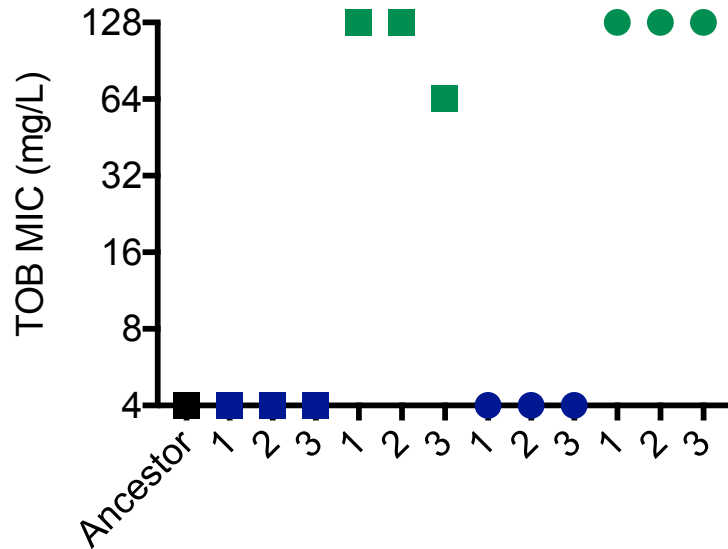

## Mueller Hinton Broth

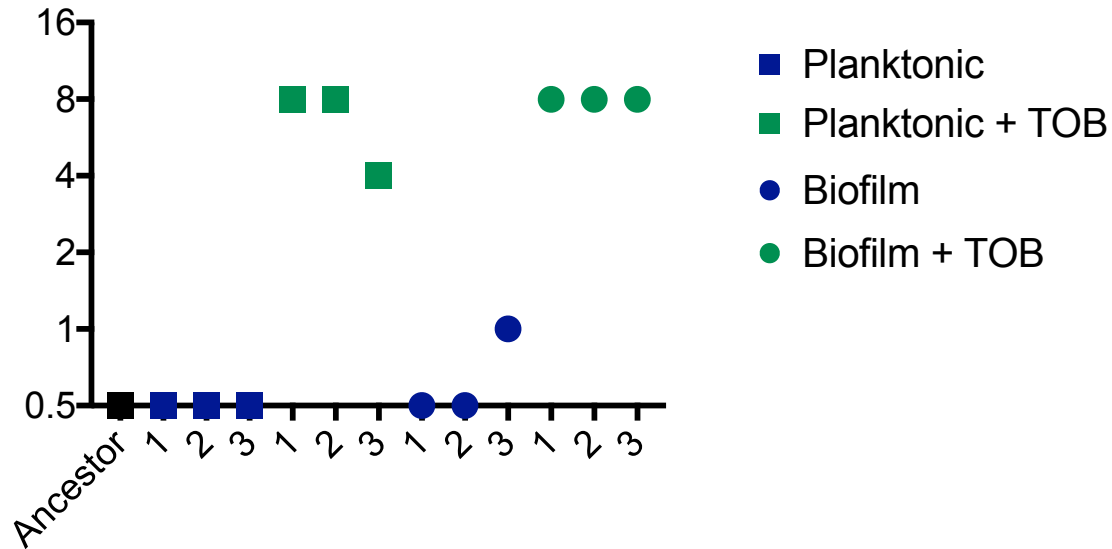

Supplement: FIG S1 [file mBio.00932-20-sf001.pdf]

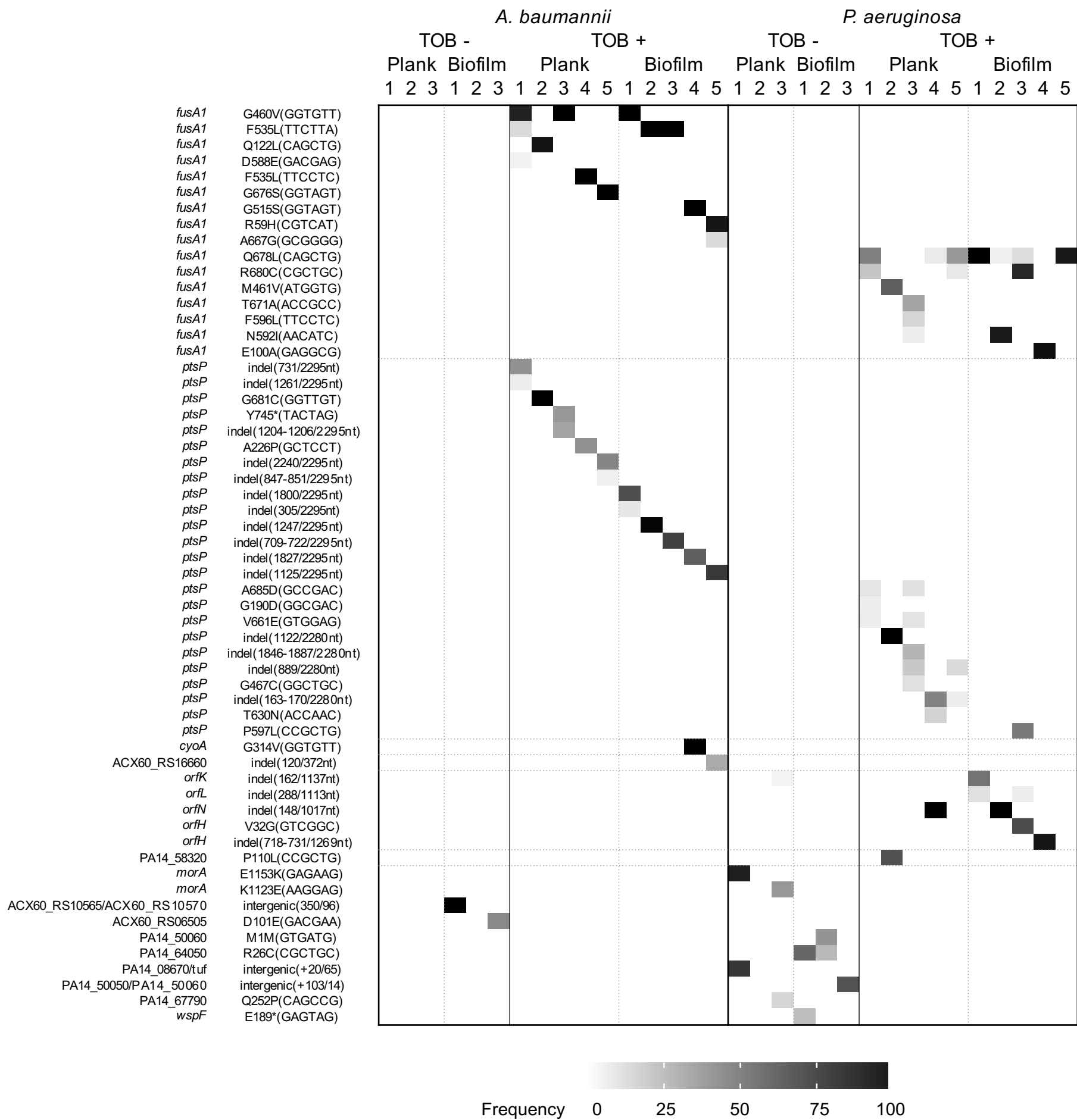

Supplement: FIG S2 [file mBio.00932-20-sf002.pdf]

***A. baumannii***

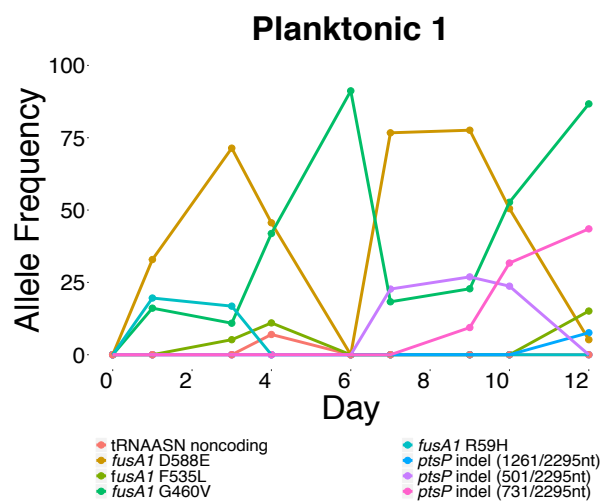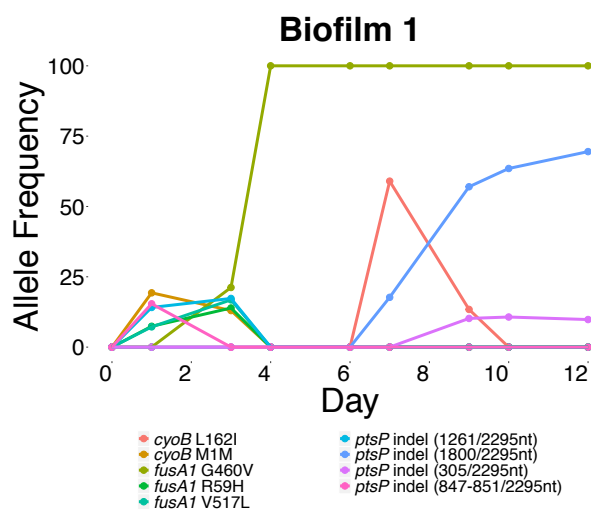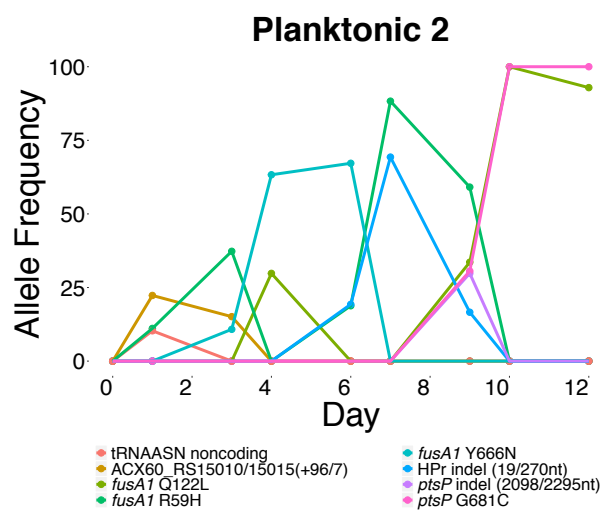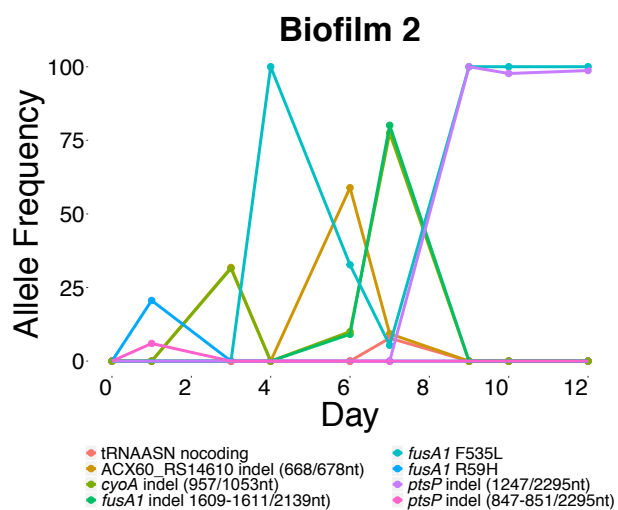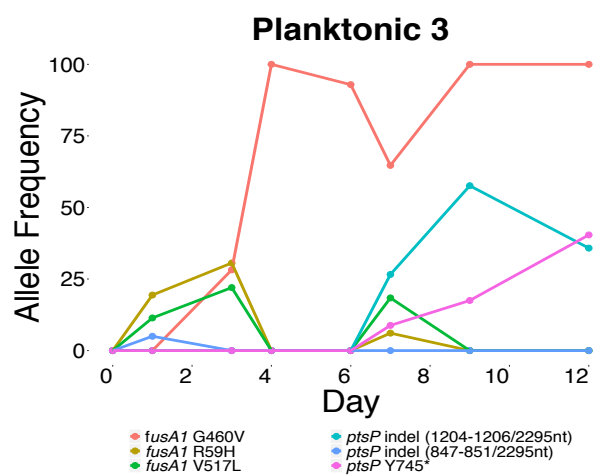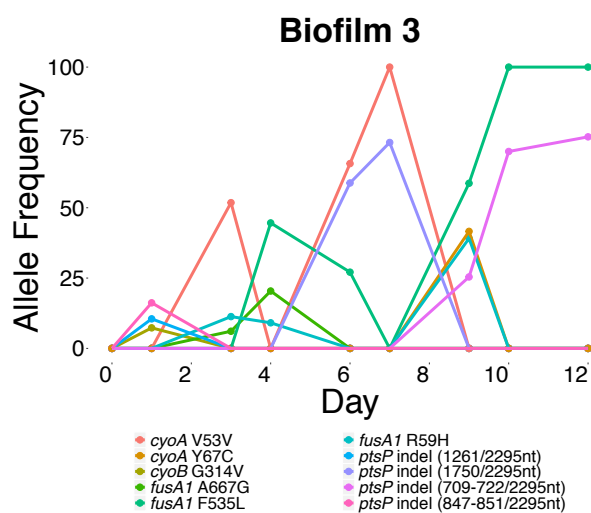

B

*P. aeruginosa*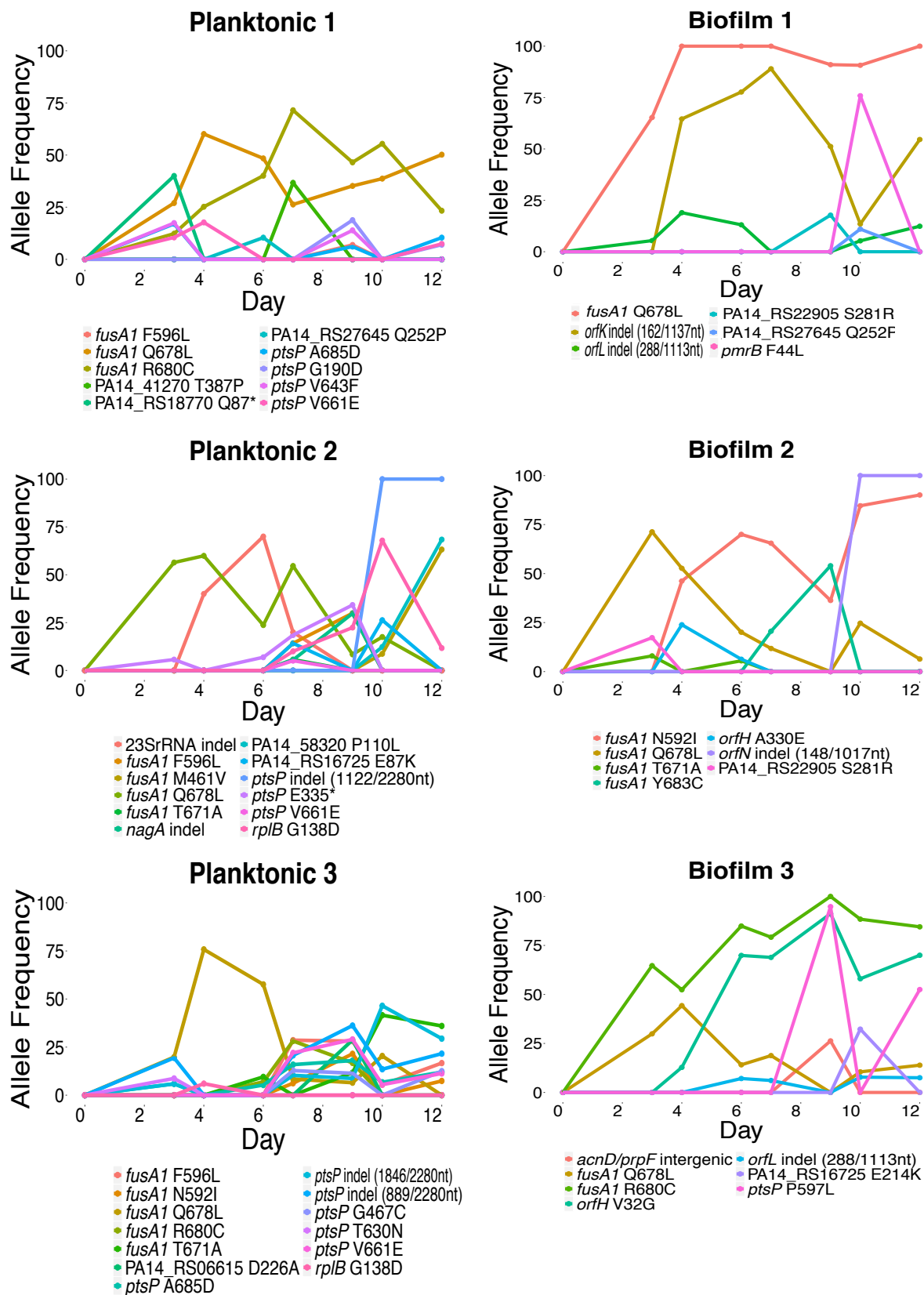

Supplement: FIG S4 [file mBio.00932-20-sf004.pdf]
