# Supplementary material for: Parallel Evolution of Tobramycin Resistance across Species and Environments
Source: mBio. 2020 May 26;11(3):e00932-20. doi: 10.1128/mBio.00932-20 (PMC7251211; doi:10.1128/mBio.00932-20)
Supplement: FIG S3 [file mBio.00932-20-sf003.pdf]

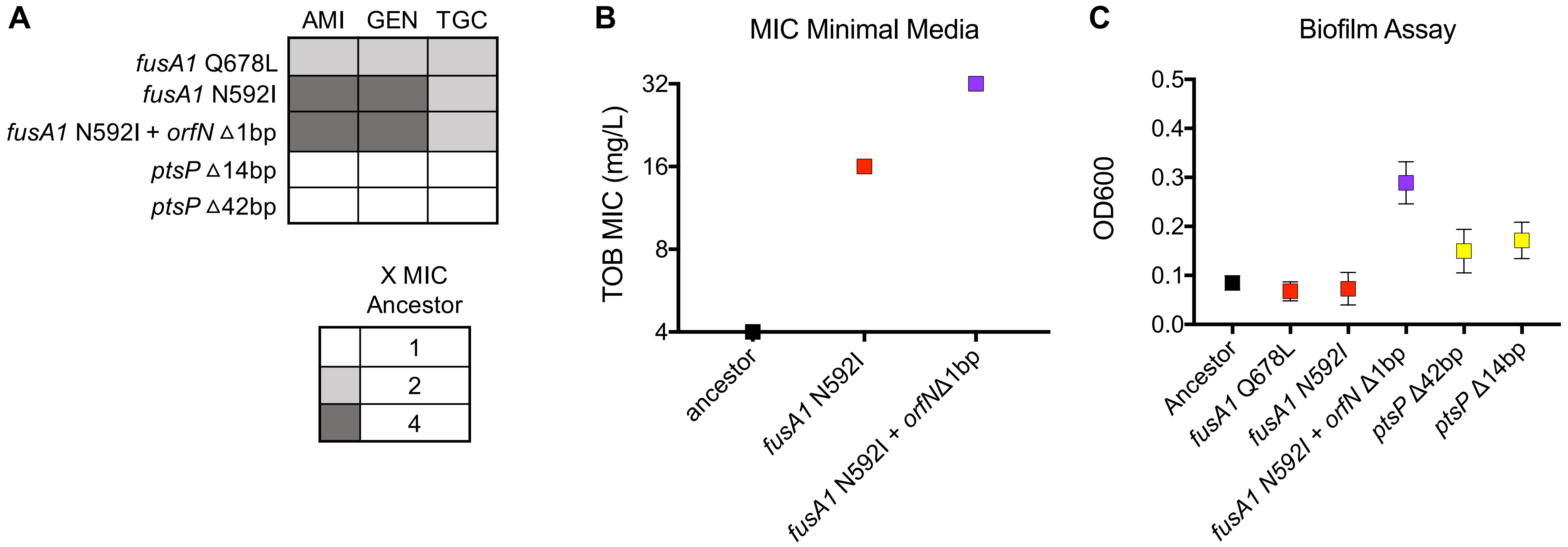
A
AMI
GEN
TGC
fusA1 Q678L
fusA1 N592I
fusA1 N592I + orfN Δ1bp
ptsP Δ14bp
ptsP Δ42bp
X MIC Ancestor
1
2
4
B
MIC Minimal Media
TOB MIC (mg/L)
32
16
8
4
ancestor
fusA1 N592I
fusA1 N592I + orfNΔ1bp
C
Biofilm Assay
OD600
0.5
0.4
0.3
0.2
0.1
0.0
Ancestor
fusA1 Q678L
fusA1 N592I
fusA1 N592I + orfN Δ1bp
ptsP Δ42bp
ptsP Δ14bp
