# Supplementary material for: Parallel Evolution of Tobramycin Resistance across Species and Environments
Source: mBio. 2020 May 26;11(3):e00932-20. doi: 10.1128/mBio.00932-20 (PMC7251211; doi:10.1128/mBio.00932-20)
Supplement: FIG S5 [file mBio.00932-20-sf005.pdf]

A. *fusA1*

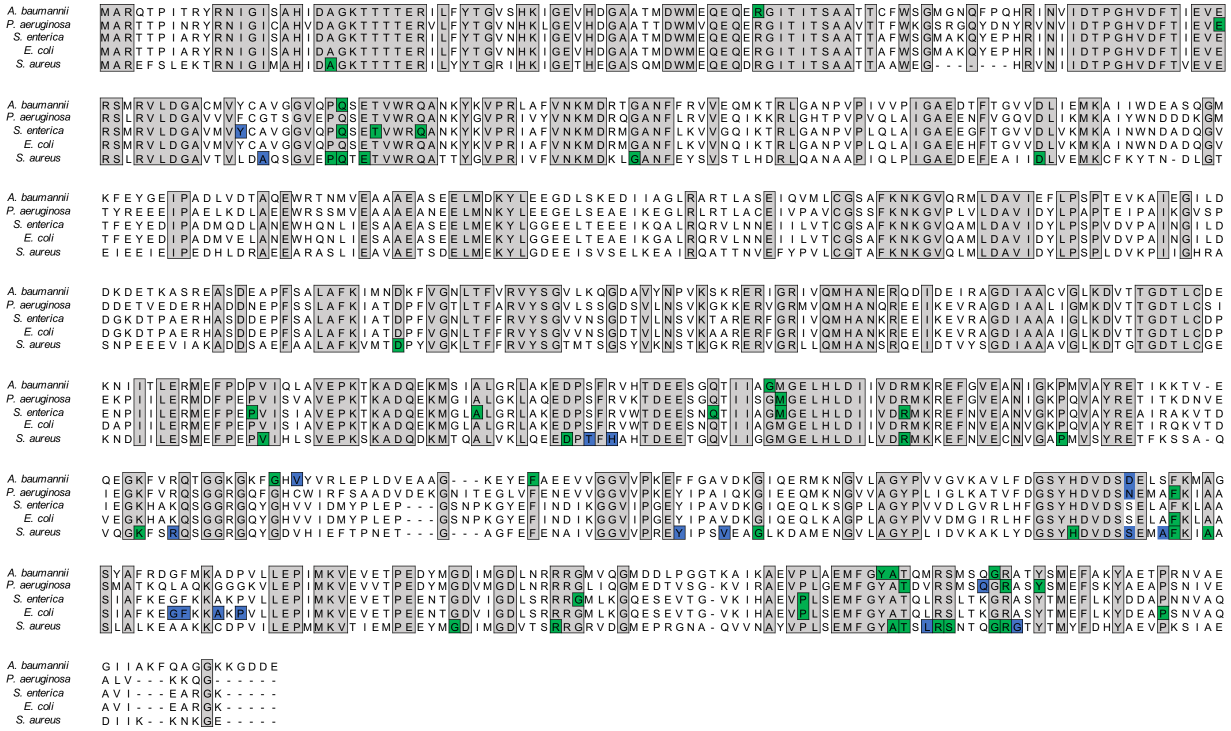

B. *ptsP*

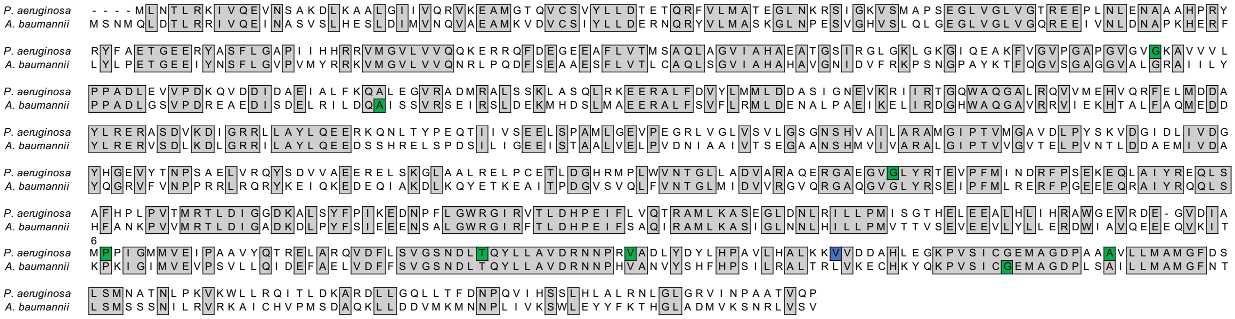

C. *cyoA*

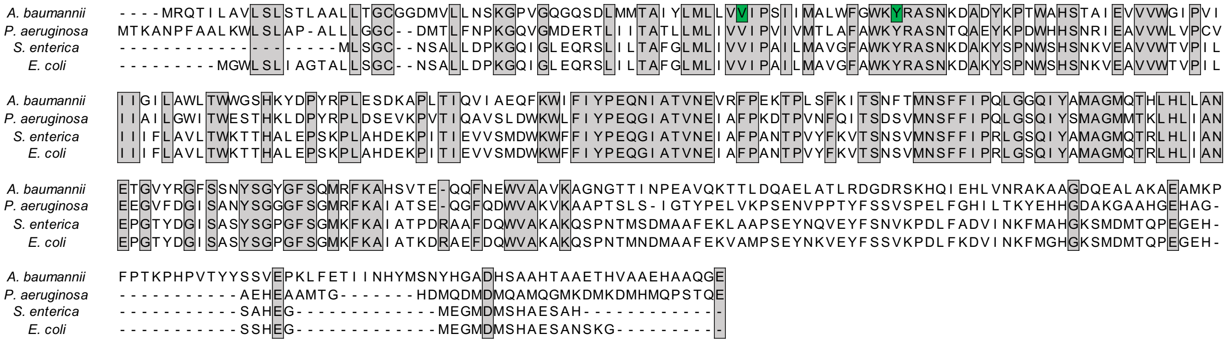

D. *cyoB*

```
A. baumannii   MDMIFGKLGWDSIP-TEPIVLVTMVFMALGAIAVLGGITYFKKWGYLWKEWFTTVDHKKIGIMYIIVSVVMLLRGFADAIMMRLQLFLAKGGGEGYLHPD
P. aeruginosa  ---MFGKLTLSAVPYHEPIVMVTLAVVALLGLGVVGAITYYRKWTYLWTEWLTSVDHKKIGVMYIVVALVMLVRGFADAIMMRGQLALAEGANHGYLPPE
S. enterica    ---MFGKLSLDAVPFHEPIVMVTIAAIIVGGLAILAAITYFGKWTYLWKEWLTSVDHKRLGIMYIIVAIVMLLRGFADAIMMRSQQALASAGEAGFLPPH
E. coli        ---MFGKLSLDAVPFHEPIVMVTIAGIILGGLALVGLITYFGKWTYLWKEWLTSVDHKRLGIMYIIVAIVMLLRGFADAIMMRSQQALASAGEAGFLPPH

A. baumannii   HYDQIFTAHGVIMIFFVAMGLVVGMMNISVPLQIGARDVAFPLLNSLSFWLFAGAAGLMMLSLVLGEFAATGWMAYPPLSGIQYSPGVGVDYYIWALQVS
P. aeruginosa  HYDQIFTAHGVIMIIFMAMPFMTGLMNLAVPLQIGARDVAFPFLNSLSFWLLVVSAMLVNVSLGLGEFARTGWVAYPPLSELAYSPGVGVDYYIWALQIS
S. enterica    HYDQIFTAHGVIMIFFVAMPFVIGLMNLVVPLQIGARDVAFPFLNNLSFWFTVVGVILVNLSLGVGEFAQTGWLAYPPLSGIEYSPSVGVDYWIWALQLS
E. coli        HYDQIFTAHGVIMIFFVAMPFVIGLMNLVVPLQIGARDVAFPFLNNLSFWFTVVGVILVNVSLGVGEFAQTGWLAYPPLSGIEYSPGVGVDYWIWSLQLS

A. baumannii   GLGTLLSGVNFFVTIIKMRAPGMKLMDMPIFTWTSLCTAVLIIASFPVLTGTLAMLTLDRYFGFHFFTNELGGSPMLYVNLIWTWGHPEVYILVLPAFGL
P. aeruginosa  GMGTLLTGINFLVTVFKMRAPGMKLMQMPIFTWTCTFANILIVASFPILTAALGLLSLDRYLDMHFFTNELGGNAMMYINLFWAWGHPEVYILILPAFGI
S. enterica    GIGTTLTGINFFVTILKMRAPGMTMFKMPVFTWASLCANVLIIASFPILTVTVALLTLDRYLGTHFFTNDMGGNMMMYINLIWAWGHPEVYILILPVFGV
E. coli        GIGTTLTGINFFVTILKMRAPGMTMFKMPVFTWASLCANVLIIASFPILTVTVALLTLDRYLGTHFFTNDMGGNMMMYINLIWAWGHPEVYILILPVFGV

A. baumannii   YSEIVATFSRKALFGYKSMVYATIAITVLAFVVWLHHFFTMGAGANVNAFFGIMTMVIAIPTGVKIFSWLFTMYKGRITFTTPMLWTLGFLVTFGIGGLT
P. aeruginosa  FSEVTATFAGKRMFGYKSMVWASAAITFLGFTVWLHHFFTMGSGGDVNGFFGVATMLISIPTGVKLFNWLFTIYKGRLRFSTPILWTLGFMVTFTIGGMT
S. enterica    FSEIAATFSRKRLFGYTSLVWATVCITVLSFIVWLHHFFTMGAGANVNAFFGITTMIIAIPTGVKIFNWLFTMYQGRIVFHSAMMWTIGFIVTFSVGGMT
E. coli        FSEIAATFSRKRLFGYTSLVWATVCITVLSFIVWLHHFFTMGAGANVNAFFGITTMIIAIPTGVKIFNWLFTMYQGRIVFHSAMLWTIGFIVTFSVGGMT

A. baumannii   GVLMAVPPADFLVHNSLFLIAHFHNVIIGGVVFGMFAGIIYYWPKMFGWKLNEAWGKAAFWFWFFGFYFAFMPLYILGFMGMTRRLNTYDNPEWDPYLAI
P. aeruginosa  GVLLAIPGADFLLHNSLFLIAHFHNTIIGGAVFGYLAGFAFWFPKAFGFTLDEKWGKRSFWCWLVGFYMAFMPLYILGFMGMTRRLNHYDNPLWKPYLVV
S. enterica    GVLLAVPGADFVLHNSLFLIAHFHNVIIGGVVFGCFAGMTYWWPKAFGFKLNETWGKRAFWFWIIGFFVAFMPLYVLGFMGMTRRLSQQIDPQFHTMLMV
E. coli        GVLLAVPGADFVLHNSLFLIAHFHNVIIGGVVFGCFAGMTYWWPKAFGFKLNETWGKRAFWFWIIGFFVAFMPLYALGFMGMTRRLSQQIDPQFHTMLMI

A. baumannii   ALFGAVLVAIGIACFLMQIIVGFLQRHQNMDYTGDPWDARTLEWATSSPAPFYNFAHEPDASGIDRFWTDKENGVAYARNTKYEDIHMPTDRAAGFVIAM
P. aeruginosa  AFFGAVLIFCGIACQLIQLFVSVRNRKQLADVNGDPWEGRTLEWATSSPPPFYNFAELPKVQDVDAFHDMKKAGTAYRKLPAYQPIHMPKNTAAGFSIAV
S. enterica    AAAGAALIALGILCQLIQIFVSIRDRDQNRDLTGDPWGGRTLEWSTSSPPPFYNFAVVPHVHERDAFWEMKEKGEAYQQPGQYEEIHMPKNSGAGIVIAA
E. coli        AASGAVLIALGILCLVIQMYVSIRDRDQNRDLTGDPWGGRTLEWATSSPPPFYNFAVVPHVHERDAFWEMKEKGEAYKKPDHYEEIHMPKNSGAGIVIAA

A. baumannii   FITLLGFALIWHIWWLVVVSFVAAVVSLIVSSFTKNVDYYVPAAEVERIENERYALLEKHLKKD--
P. aeruginosa  FAFVFGFAAIWHIWWLMAVGFVGMIGSFIVRSYNQDVDYYVQPEEIEKIESARFQQLAKQV-----
S. enterica    FATVFGFAMIWHIWWLAIVGFAGMIISWIVKSFDEDVDYYVPVPEVEKLENQHFDEITKAGLKNGN
E. coli        FSTIFGFAMIWHIWWLAIVGFAGMIITWIVKSFDEDVDYYVPVAEIEKLENQHFDEITKAGLKNGN
```
